# Twenty-Seven Years of Phase III Trials for Patients with Extensive Disease Small-Cell Lung Cancer: Disappointing Results

**DOI:** 10.1371/journal.pone.0007835

**Published:** 2009-11-13

**Authors:** Isao Oze, Katsuyuki Hotta, Katsuyuki Kiura, Nobuaki Ochi, Nagio Takigawa, Yoshiro Fujiwara, Masahiro Tabata, Mitsune Tanimoto

**Affiliations:** Department of Respiratory Medicine, Okayama University Hospital, Okayama, Japan; City of Hope National Medical Center, United States of America

## Abstract

**Background:**

Few studies have formally assessed whether treatment outcomes have improved substantially over the years for patients with extensive disease small-cell lung cancer (ED-SCLC) enrolled in phase III trials. The objective of the current investigation was to determine the time trends in outcomes for the patients in those trials.

**Methods and Findings:**

We searched for trials that were reported between January 1981 and August 2008. Phase III randomized controlled trials were eligible if they compared first-line, systemic chemotherapy for ED-SCLC. Data were evaluated by using a linear regression analysis. Results: In total, 52 trials were identified that had been initiated between 1980 and 2006; these studies involved 10,262 patients with 110 chemotherapy arms. The number of randomized patients and the proportion of patients with good performance status (PS) increased over time. Cisplatin-based regimens, especially cisplatin and etoposide (PE) regimen, have increasingly been studied, whereas cyclophosphamide, doxorubicin, and vincristine–based regimens have been less investigated. Multiple regression analysis showed no significant improvement in survival over the years. Additionally, the use of a PE regimen did not affect survival, whereas the proportion of patients with good PS and the trial design of assigning prophylactic cranial irradiation were significantly associated with favorable outcome.

**Conclusions and Significance:**

The survival of patients with ED-SCLC enrolled in phase III trials did not improve significantly over the years, suggesting the need for further development of novel targets, newer agents, and comprehensive patient care.

## Introduction

Lung cancer is a leading cause of cancer-related mortality in many industrialized countries. Small-cell lung cancer (SCLC), which accounts for about 15% of all lung cancer cases, is categorized into two clinical stages: limited disease (LD) and extensive disease (ED). For patients with ED-SCLC, combination chemotherapy is the mainstay of treatment.

In the 1980s, the most widely used combination of drugs for initial treatment of ED-SCLC was cyclophosphamide, doxorubicin, and vincristine (CAV), which produced a median survival time of 9 to 11 months [1]. In the late 1980s, a combination regimen of cisplatin and etoposide (PE) was introduced, and an alternating regimen of PE and CAV has been widely investigated in randomized controlled trials [2].

In 1999, the results of a systemic review indicated a modest improvement over the years in the survival time of patients with ED-SCLC treated with chemotherapy between 1972 and 1994 [3]. This improvement was potentially attributable to (i) introduction of the PE regimen in the late 1980s and (ii) improvements in the supportive care and general management of the patients. However, this included just North American trials and would provide some justification for looking at the world-wide result.

A decade has passed since that systemic review, and recent clinical trials have investigated newer antineoplastic agents such as irinotecan and topotecan. Thus, we performed a literature search to determine whether patient outcomes have improved in the treatment of ED-SCLC.

## Materials and Methods

### Searching

We searched for trials that were reported between January 1981 and August 2008. To avoid publication bias, we identified both published and unpublished trials through a computer-based search of the PubMed database and abstracts from past conferences of the American Society of Clinical Oncology (1998–2008). We used the following search terms: *lung neoplasm*, *carcinoma*, *small-cell*, *chemotherapy*, and *randomized controlled trial*. The search was guided by a thorough examination of reference lists from original articles, review articles, relevant books, and the Physician Data Query registry of clinical trials.

### Selection

Phase III randomized controlled trials were eligible for inclusion in this study if they compared first-line, systemic chemotherapy for ED-SCLC that contained cytotoxic agents, providing the year of trial initiation. Trials were excluded if they only investigated immunotherapy regimens, or if they enrolled only responders to the initial chemotherapy. Trials initially designed to assess combined-modality treatment, including radiotherapy and surgery concurrently undergone with the initial chemotherapy, were also ineligible, but those optionally designed to conduct these therapies or prophylactic cranial irradiation (PCI) sequentially after the induction chemotherapy were allowed. Some phase III trials incorporated patients with both LD-SCLC and ED-SCLC. These were considered eligible only if survival data for patients with ED-SCLC could be solely obtained. We acknowledge that the definitions for LD-SCLC and ED-SCLC vary somewhat in the different groups compared, and we could not strictly reallocate each patient because we were unable to access the individual patient databases. Instead, we applied the definition described in each original report to this study. If no relevant descriptions were documented, we considered that the definition in that trial would have been based on the guidelines in existence at the time of that trial initiation [4], [5]. The control arms in each of the phase III trials were identified based on statements in each trial.

### Validity Assessment

To avoid bias in the data abstraction process, four medical oncologists (I.O., N.O., Y.F., and K.H.), one of whom (K.H.) holds a board certificate for medical oncology, independently abstracted the data from the trials and subsequently compared the results. All data were checked for internal consistency, and disagreements were resolved by discussion among the investigators.

### Data Abstraction

The following information was obtained from each report: year of trial initiation (i.e., year when the first patient was accrued); number of patients enrolled and randomized; median age of patients; proportion of patients with good performance status (PS); proportion of patients who were male and who had brain metastasis; chemotherapy regimen; definition of ED; description of the administration of sequential thoracic irradiation, surgery, or PCI as one of the trial designs; and median survival time (per treatment arm).

### Study Characteristics

All studies included were phase III randomized controlled trials of first-line systemic chemotherapy for ED-SCLC. The study outcomes were median survival time. Variation in study characteristics and clinical heterogeneity between studies were adjusted statistically (see below).

### Quantitative Data Synthesis

Data from phase III trials were evaluated by using multiple, stepwise regression analysis (with the following stepping method criteria: probability of F to enter the model, <0.05; to remove from the model, >0.10). The data analyzed included year of trial initiation, use of PE regimen, maximal age of patients, proportion of patients with good PS, proportion of male patients, and definition of PCI settings. These data were used to determine whether each factor had an independent impact on the survival of patients with ED-SCLC who were treated in the phase III studies over time. All *P* values corresponded to 2-sided tests, and significance was set at *P*<0.05.

## Results

### Trial Flow/Flow of Included Studies

**Figure 1:**
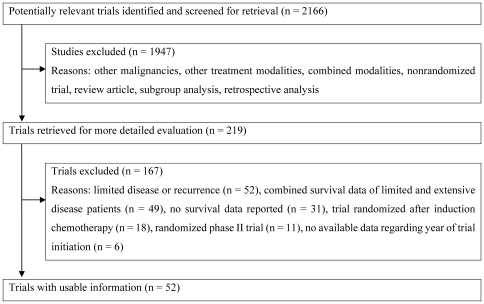
Flow chart showing the progress of trials through the review.

Figure 1 shows a flow chart of this study. In total, 52 trials for ED-SCLC were identified as a result of the computer-based and manual searches for relevant articles, abstracts, and references (Please see File S1). A total of 10,262 patients had been allocated randomly to 110 chemotherapy arms.

### Study Characteristics

**Figure 2:**
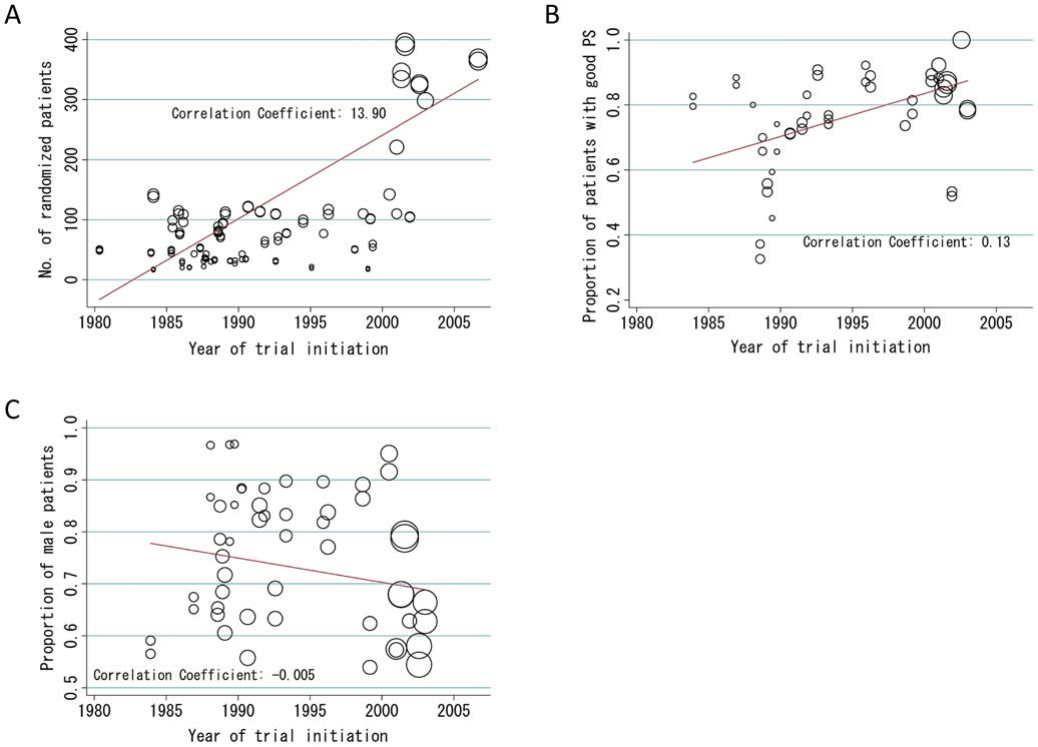
Trends in trial characteristics. These charts show the associations between year of trial initiation and number of randomized patients (A), proportion of patients with good PS (B), and proportion of male patients (C) in each trial. The size of solid circles represents data weighted on the basis of the number of randomized patients. Abbreviations: PS, performance status.

Table 1 lists the baseline characteristics of the trials. Trials were initiated between 1980 and 2006. The number of randomized patients and the proportion of patients with good PS increased over time (13.9 patient increase/year, *P*<0.001; and 1.32% increase/year, *P*<0.001, respectively; Figures 2A and 2B), whereas the proportion of male patients remained consistent (0.47% decrease/year, *P* = 0.114; Figure 2C). In 19 trials that assigned PCI, it was planned that patients who achieved a complete response (CR) or CR/partial response (PR) after induction chemotherapy would receive PCI. Thirteen (25%) of the 52 phase III trials showed a statistically significantly difference in survival time. Of these, eight were in favor of the patient cohort that received the experimental therapy compared with the control group, while the remaining five were in favor of that in the control group.

**Table 1:** Characteristics of the 52 Randomized Trials.

Variable		Value
No. of trials		52
	(No. of randomized patients in all trials 10262)	
No. of treatment arms		
	2	47
	3	4
	4	1
Year of trial initiation		
	Median (range)	1990 (1980–2006)
No. of randomized patients (%)		
	<100	35
	100–200	25
	200–300	29
	>300	11
	Median (range)	158 (34–786)
Proportion of patients with good performance status† (%)		
	<80	50
	80–90	42
	>90	8
	Median percentage (range)	80 (35–100)
Male Patients (%)		
	<80	54
	80–90	35
	>90	11
	Median percentage (range)	75 (56–93)
Trials assigning PCI for those with CR or CR/PR to the initial chemotherapy		
	Yes	37
	No	63
Trials with a statistically significant difference in overall survival time (%)		
	Yes	25
	No	65
	Not recorded	10

†Defined as a performance status of 0 or 1.

Abbreviations; PCI, prophylactic cranial irradiation; CR, complete response; PR, pertial response.

### Types of Chemotherapy Arms

There were 110 chemotherapy treatment arms in the 52 phase III trials (Table 2). Cisplatin-based regimens were the most frequently investigated. The PE regimen, currently considered as the standard treatment for patients with ED-SCLC, has increasingly been studied (Figure 1). As expected, the CAV alternating PE regimen was extensively examined in the 1980s, but this decreased in the 1990s.

**Table 2:** Types of Chemotherapy Arms and Treatment Outcomes (Per Treatment Arm).

Chemotherapy Arm					No. of Arms (%)	MST [range], months	
Total no. of arms					110	9.3	[4.9–14.5]
Platinum-based regimens					78 (70.9)	9.5	[4.9–14.5]
	Cisplatin-based				64 (58.2)	9.6	[5.8–14.5]
		CAV alternating PE			16 (14.5)	9.5	[5.8–14.5]
		PE			16 (14.5)	9.4	[7.0–10.2]
		Other Cisplatin-based			32 (29.1)	9.8	[6.7–12.8]
Nonplatinum regimens					32 (29.1)	8.5	[5.0–13.0]
	CAV-based				10 (9.1)	9.1	[7.5–13.8]
	Non-CAV-based combination therapy				19 (17.3)	8.2	[5.0–13.0]
	Non-CAV-based monotherapy				3 (2.7)	8.3	[6.0–9.3]

Abbreviations: MST, median survival time; CAV, cyclophosphamide, doxorubicin, and vincristine; PE, cisplatin and etoposide.

### Trends in Patient Survival

**Figure 3:**
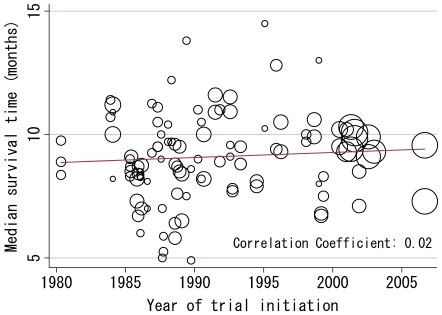
Relationship between year of trial initiation and median survival time. Analysis was weighted by the number of randomized patients. Each trial is represented by a circle; the size of each circle is proportional to the sample size of randomized patients in the given trial.

Data on patient survival were available from all 52 trials and 110 chemotherapy arms and analyzed by treatment arm. A scattergram of the two parameters (year of trial initiation and median survival time) revealed that the slope of the fitted line was 0.021, indicating a 0.021 month (0.63 day) increase in median survival time per year (*P* = 0.272; Figure 3). Multiple regression analysis, adjusting for several confounding trial characteristics, also showed no significant association between the two parameters (regression coefficient for year of trial initiation  = 0.011, 95% confidence interval = −0.36–0.38, *P* = 0.950; Table 3). In this setting, the proportion of patients with good PS was significantly associated with a favorable outcome. The multiple regression analysis also showed a significant influence of PCI setting on survival prolongation. This finding is partly supported by a recent report on the survival advantage of PCI in ED-SCLC patients who responded to initial chemotherapy [6].

**Table 3:** Multiple Stepwise Linear Regression Analysis of Overall Survival (Per Treatment Arm).

Factor	Regression Coefficient*	SE	*P* †
Year of trial initiation	Excluded		
Use of PE regimen (y or n)	Excluded		
Proportion of patients with good PS	6.65	1.30	<0.001
Proportion of male patients	Excluded		
Median age of patients	Excluded		
Design of the PCI setting (y or n)	2.14	0.742	0.009
Description of definition for ED (y or n)	Excluded		

*Threshold F values for entering and removing from the model were 0.05 and 0.10, respectively.

†*P*<0.05 was considered significant. This multivariate stepwise regression model excluded the factors “Year of trial initiation,” “Use of PE regimen,” “Proportion of male patients,” “Median age of patients,” and “Description of definition for ED” from the model.

Abbreviations: PE, cisplatin and etoposide; PS, performance status; PCI, prophilactic cranial irradiation; ED, extended disease.

## Discussion

Our results demonstrate no significant improvement in patient outcomes over the years in phase III trials of systemic chemotherapy for ED-SCLC, with an increase of 0.021 months (0.63 days) per year (univariate analysis; *P* = 0.272; Figure 3) confirmed in the multivariate model (*P* = 0.950; Table 3). However, the proportion of patients with good PS and the trial design of assigning PCI for those with CR or CR/PR significantly influenced survival (Table 3).

The introduction of multiple drug regimens has been a great advance in the treatment of ED-SCLC; indeed, the CAV regimen yielded a survival time approximately twice as long as that of the single-agent therapy frequently used in the early 1970s [1], [7]. However, the survival benefit from chemotherapy has reached somewhat of a plateau, even with the introduction of the PE regimen in recent clinical trials, as compared with the CAV regimen or CAV alternating PE [2], [8], [9], [10]. In addition, most of newer antitumour agents introduced after PE (e.g., irinotecan and topotecan) failed to substantially prolong survival in the first-line setting over the standard PE regimen [11], [12], [13], [14], [15]. Thus, based on these findings, our main results demonstrate no significant improvement in survival since 1980. In contrast, a 1999 study showed a significant increase in overall survival time [3]. This difference in the time trend in overall survival is mainly attributable to differences in the study period (year of trial initiation: 1972–1994 vs. 1980–2006 in the earlier and present study, respectively; [3]).

In Figure 3, trials between 2000 and 2005 appeared to show extensive clustering with median survival time of around ten months. It would be attributable to some common characteristics among these trials, such as relatively uniformed chemotherapeutic regimens (cisplatin-based ones) and larger number of the registered patients. In contrast, there were other trial arms that yielded the longest versus shortest survival times (14–15 months versus 5–6 months). These included less number of the enrolled patients, which possibly resulted in a wide-range distribution in the Figure.

We investigated a similar issue previously [16], namely trends in prognosis over the years in chemo-naïve patients with advanced non-small cell lung cancer (NSCLC) enrolled in phase III trials. The analysis similarly revealed a very small increase in patient survival (3.61 days per year) but one that was statistically significant in the multiple regression model (*P*<0.001; ([16]). There may be several potential factors behind such differences in statistical results in SCLC and NSCLC settings. The most important is that new active agents such as taxanes appeared in the treatment of NSCLC [17], [18] and few novel agents, including molecular-targeted agents, did in the treatment for SCLC [11], [19], [20], [21] in these study periods. Another hypothesis is that advanced NSCLC might be more influenced than SCLC by lead time bias through early detection with improved imaging techniques, mainly because the growth rate of NSCLC is generally less rapid than that of SCLC throughout its natural history [22]. Progress in supportive care practices would lead to improvements in survival among patients with advanced NSCLC. Those with advanced NSCLC usually have less rapid disease progression and, thus, would likely benefit from its advancement. Finally, the statistical difference between our NSCLC and SCLC studies could have arisen from differences in sample size (number of trials), indicating that the current study may have lacked adequate power to accurately evaluate the association between the year of trial initiation and patient outcome.

The potential influence of second-line chemotherapy should also be considered in assessing the effect of first-line chemotherapy because it may contribute to recent improvements in survival [23]. The trials analyzed here rarely provided information about second-line treatment, and we can not assess its exact effect in this setting. There are few positive phase III trials of second-line treatments, and thus it is unlikely that such therapy can significantly confound patient prognosis after the initiation of first-line chemotherapy [24].

In conclusion, the results of our analysis suggest that, regardless of the reason, the survival of patients with ED-SCLC who were enrolled in phase III trials did not improve significantly over the years. Thus, the development of novel targets, newer agents, and comprehensive patient care will be essential in the future fight against lung cancer.

## Supporting Information

File S1 (0.05 MB DOC)
