# Supplementary material for: Twenty-Seven Years of Phase III Trials for Patients with Extensive Disease Small-Cell Lung Cancer: Disappointing Results
Source: PLoS One. 2009 Nov 13;4(11):e7835. doi: 10.1371/journal.pone.0007835 (PMC2773043; doi:10.1371/journal.pone.0007835)
Supplement: File S1 — (0.05 MB DOC) [file pone.0007835.s001.doc]

**References included in this study**

1. Pirker R, Ramlau RA, Schuette W et al. Safety and efficacy of darbepoetin alpha in previously untreated extensive-stage small-cell lung cancer treated with platinum plus etoposide. J Clin Oncol 2008; 26: 2342-2349.

2. Baka S, Califano R, Ferraldeschi R et al. Phase III randomised trial of doxorubicin-based chemotherapy compared with platinum-based chemotherapy in small-cell lung cancer. Br J Cancer 2008; 99: 442-447.

3. Okamoto H, Watanabe K, Kunikane H et al. Randomised phase III trial of carboplatin plus etoposide vs split doses of cisplatin plus etoposide in elderly or poor-risk patients with extensive disease small-cell lung cancer: JCOG 9702. Br J Cancer 2007; 97: 162-169.

4. de Jong WK, Groen HJ, Koolen MG et al. Phase III study of cyclophosphamide, doxorubicin, and etoposide compared with carboplatin and paclitaxel in patients with extensive disease small-cell lung cancer. Eur J Cancer 2007; 43: 2345-2350.

5. Reck M, von Pawel J, Macha HN et al. Efficient palliation in patients with small-cell lung cancer by a combination of paclitaxel, etoposide and carboplatin: quality of life and 6-years'-follow-up results from a randomised phase III trial. Lung Cancer 2006; 53: 67-75.

6. Hanna N, Bunn PA, Jr., Langer C et al. Randomized phase III trial comparing irinotecan/cisplatin with etoposide/cisplatin in patients with previously untreated extensive-stage disease small-cell lung cancer. J Clin Oncol 2006; 24: 2038-2043.

7. Eckardt JR, von Pawel J, Papai Z et al. Open-label, multicenter, randomized, phase III study comparing oral topotecan/cisplatin versus etoposide/cisplatin as treatment for chemotherapy-naive patients with extensive-disease small-cell lung cancer. J Clin Oncol 2006; 24: 2044-2051.

8. Ignatiadis M, Mavroudis D, Veslemes M et al. Sequential versus alternating administration of cisplatin/etoposide and topotecan as first-line treatment in extensive-stage small-cell lung cancer: preliminary results of a Phase III Trial of the Hellenic Oncology Research Group. Clin Lung Cancer 2005; 7: 183-189.

9. Artal-Cortes A, Gomez-Codina J, Gonzalez-Larriba JL et al. Prospective randomized phase III trial of etoposide/cisplatin versus high-dose epirubicin/cisplatin in small-cell lung cancer. Clin Lung Cancer 2004; 6: 175-183.

10. Altinbas M, Coskun HS, Er O et al. A randomized clinical trial of combination chemotherapy with and without low-molecular-weight heparin in small cell lung cancer. J Thromb Haemost 2004; 2: 1266-1271.

11. Sundstrom S, Bremnes RM, Kaasa S et al. Cisplatin and etoposide regimen is superior to cyclophosphamide, epirubicin, and vincristine regimen in small-cell lung cancer: results from a randomized phase III trial with 5 years' follow-up. J Clin Oncol 2002; 20: 4665-4672.

12. Noda K, Nishiwaki Y, Kawahara M et al. Irinotecan plus cisplatin compared with etoposide plus cisplatin for extensive small-cell lung cancer. N Engl J Med 2002; 346: 85-91.

13. Sculier JP, Paesmans M, Lecomte J et al. A three-arm phase III randomised trial assessing, in patients with extensive-disease small-cell lung cancer, accelerated chemotherapy with support of haematological growth factor or oral antibiotics. Br J Cancer 2001; 85: 1444-1451.

14. Pujol JL, Daures JP, Riviere A et al. Etoposide plus cisplatin with or without the combination of 4'-epidoxorubicin plus cyclophosphamide in treatment of extensive small-cell lung cancer: a French Federation of Cancer Institutes multicenter phase III randomized study. J Natl Cancer Inst 2001; 93: 300-308.

15. Hirsch FR, Osterlind K, Jeppesen N et al. Superiority of high-dose platinum (cisplatin and carboplatin) compared to carboplatin alone in combination chemotherapy for small-cell lung carcinoma: a prospective randomised trial of 280 consecutive patients. Ann Oncol 2001; 12: 647-653.

16. Masutani M, Ochi Y, Kadota A et al. Dose-intensive weekly alternating chemotherapy for patients with small cell lung cancer: randomized trial, can it improve survival of patients with good prognostic factors? Oncol Rep 2000; 7: 305-310.

17. Lai SL, Perng RP. Combination versus alternating chemotherapy in small-cell lung cancer. Zhonghua Yi Xue Za Zhi (Taipei) 2000; 63: 513-520.

18. Urban T, Baleyte T, Chastang CL et al. Standard combination versus alternating chemotherapy in small cell lung cancer: a randomised clinical trial including 394 patients. 'Petites Cellules' Group. Lung Cancer 1999; 25: 105-113.

19. Murray N, Livingston RB, Shepherd FA et al. Randomized study of CODE versus alternating CAV/EP for extensive-stage small-cell lung cancer: an Intergroup Study of the National Cancer Institute of Canada Clinical Trials Group and the Southwest Oncology Group. J Clin Oncol 1999; 17: 2300-2308.

20. Wood L, Palmer M, Hewitt J et al. Results of a phase III, double-blind, placebo-controlled trial of megestrol acetate modulation of P-glycoprotein-mediated drug resistance in the first-line management of small-cell lung carcinoma. Br J Cancer 1998; 77: 627-631.

21. Ueoka H, Kiura K, Tabata M et al. A randomized trial of hybrid administration of cyclophosphamide, doxorubicin, and vincristine (CAV)/cisplatin and etoposide (PVP) versus sequential administration of CAV-PVP for the treatment of patients with small cell lung carcinoma: results of long term follow-up. Cancer 1998; 83: 283-290.

22. Furuse K, Fukuoka M, Nishiwaki Y et al. Phase III study of intensive weekly chemotherapy with recombinant human granulocyte colony-stimulating factor versus standard chemotherapy in extensive-disease small-cell lung cancer. The Japan Clinical Oncology Group. J Clin Oncol 1998; 16: 2126-2132.

23. Tummarello D, Mari D, Graziano F et al. A randomized, controlled phase III study of cyclophosphamide, doxorubicin, and vincristine with etoposide (CAV-E) or teniposide (CAV-T), followed by recombinant interferon-alpha maintenance therapy or observation, in small cell lung carcinoma patients with complete responses. Cancer 1997; 80: 2222-2229.

24. Pujol JL, Douillard JY, Riviere A et al. Dose-intensity of a four-drug chemotherapy regimen with or without recombinant human granulocyte-macrophage colony-stimulating factor in extensive-stage small-cell lung cancer: a multicenter randomized phase III study. J Clin Oncol 1997; 15: 2082-2089.

25. Prior C, Oroszy S, Oberaigner W et al. Adjunctive interferon-alpha-2c in stage IIIB/IV small-cell lung cancer: a phase III trial. Eur Respir J 1997; 10: 392-396.

26. Fukuoka M, Masuda N, Negoro S et al. CODE chemotherapy with and without granulocyte colony-stimulating factor in small-cell lung cancer. Br J Cancer 1997; 75: 306-309.

27. Rowland KM, Jr., Loprinzi CL, Shaw EG et al. Randomized double-blind placebo-controlled trial of cisplatin and etoposide plus megestrol acetate/placebo in extensive-stage small-cell lung cancer: a North Central Cancer Treatment Group study. J Clin Oncol 1996; 14: 135-141.

28. Postmus PE, Scagliotti G, Groen HJ et al. Standard versus alternating non-cross-resistant chemotherapy in extensive small cell lung cancer: an EORTC Phase III trial. Eur J Cancer 1996; 32A: 1498-1503.

29. Lassen U, Kristjansen PE, Osterlind K et al. Superiority of cisplatin or carboplatin in combination with teniposide and vincristine in the induction chemotherapy of small-cell lung cancer. A randomized trial with 5 years follow up. Ann Oncol 1996; 7: 365-371.

30. James LE, Gower NH, Rudd RM et al. A randomised trial of low-dose/high-frequency chemotherapy as palliative treatment of poor-prognosis small-cell lung cancer: a Cancer research Campaign trial. Br J Cancer 1996; 73: 1563-1568.

31. Joss RA, Bacchi M, Hurny C et al. Early versus late alternating chemotherapy in small-cell lung cancer. Swiss Group for Clinical Cancer Research (SAKK). Ann Oncol 1995; 6: 157-166.

32. Joss RA, Alberto P, Hurny C et al. Quality versus quantity of life in the treatment of patients with advanced small-cell lung cancer? A randomized phase III comparison of weekly carboplatin and teniposide versus cisplatin, adriamycin, etoposide alternating with cyclophosphamide, methotrexate, vincristine and lomustine. Swiss Group for Clinical Cancer Research (SAKK). Ann Oncol 1995; 6: 41-48.

33. Souhami RL, Rudd R, Ruiz de Elvira MC et al. Randomized trial comparing weekly versus 3-week chemotherapy in small-cell lung cancer: a Cancer Research Campaign trial. J Clin Oncol 1994; 12: 1806-1813.

34. Maksymiuk AW, Jett JR, Earle JD et al. Sequencing and schedule effects of cisplatin plus etoposide in small-cell lung cancer: results of a North Central Cancer Treatment Group randomized clinical trial. J Clin Oncol 1994; 12: 70-76.

35. Joss RA, Alberto P, Bleher EA et al. Combined-modality treatment of small-cell lung cancer: randomized comparison of three induction chemotherapies followed by maintenance chemotherapy with or without radiotherapy to the chest. Swiss Group for Clinical Cancer Research (SAKK). Ann Oncol 1994; 5: 921-928.

36. Ihde DC, Mulshine JL, Kramer BS et al. Prospective randomized comparison of high-dose and standard-dose etoposide and cisplatin chemotherapy in patients with extensive-stage small-cell lung cancer. J Clin Oncol 1994; 12: 2022-2034.

37. Clark PI, Slevin ML, Joel SP et al. A randomized trial of two etoposide schedules in small-cell lung cancer: the influence of pharmacokinetics on efficacy and toxicity. J Clin Oncol 1994; 12: 1427-1435.

38. Anderson H, Hopwood P, Prendiville J et al. A randomised study of bolus vs continuous pump infusion of ifosfamide and doxorubicin with oral etoposide for small cell lung cancer. Br J Cancer 1993; 67: 1385-1390.

39. Nou E, Lamberg K, Brodin O. Etoposide versus methotrexate in small cell bronchial carcinoma. A randomized study of two types of four-drug chemotherapy regimens. Acta Oncol 1992; 31: 853-860.

40. Monnet I, Chariot P, Quoix E et al. Extensive small-cell lung cancer. A randomized comparison of two chemotherapy programs with early crossover in instances of failure. Association pour le Traitement des Tumeurs Intra-Thoraciques (ATTIT). Ann Oncol 1992; 3: 813-817.

41. Miyamoto H, Nakabayashi T, Isobe H et al. A phase III comparison of etoposide/cisplatin with or without added ifosfamide in small-cell lung cancer. Oncology 1992; 49: 431-435.

42. Gatzemeier U, von Pawel J, Laumen R et al. Carboplatin/etoposide/vincristine therapy in small cell lung cancer. Oncology 1992; 49 Suppl 1: 25-33.

43. Ettinger DS, Finkelstein DM, Abeloff MD et al. Justification for evaluating new anticancer drugs in selected untreated patients with extensive-stage small-cell lung cancer: an Eastern Cooperative Oncology Group randomized study. J Natl Cancer Inst 1992; 84: 1077-1084.

44. Wolf M, Pritsch M, Drings P et al. Cyclic-alternating versus response-oriented chemotherapy in small-cell lung cancer: a German multicenter randomized trial of 321 patients. J Clin Oncol 1991; 9: 614-624.

45. Jones AL, Holborn J, Ashley S et al. Effective new low toxicity chemotherapy with carboplatin, vinblastine and methotrexate for small cell lung cancer: a randomised trial against doxorubicin, cyclophosphamide and etoposide. Eur J Cancer 1991; 27: 866-870.

46. Johnson DH, Ruckdeschel JC, Keller JH et al. A randomized trial to compare intravenous and oral etoposide in combination with cisplatin for the treatment of small cell lung cancer. Cancer 1991; 67: 245-249.

47. Fukuoka M, Furuse K, Saijo N et al. Randomized trial of cyclophosphamide, doxorubicin, and vincristine versus cisplatin and etoposide versus alternation of these regimens in small-cell lung cancer. J Natl Cancer Inst 1991; 83: 855-861.

48. Earl HM, Rudd RM, Spiro SG et al. A randomised trial of planned versus as required chemotherapy in small cell lung cancer: a Cancer Research Campaign trial. Br J Cancer 1991; 64: 566-572.

49. Socinski M, Smit EF, Lorigan P et al. Phase III study of pemetrexed plus carboplatin (PC) versus etoposide plus carboplatin (EC) in chemonaive patients (pts) with extensive-stage disease small cell lung cancer (ED-SCLC): Interim results. Proc Am Soc Clin Oncol 2008;(abstr NSA).

50. Lara PN Jr, Natale R, Crowley J, Lenz HJ, Redman MW, Carleton JE, Jett J, Langer CJ, Kuebler JP, Dakhil SR, Chansky K, Gandara DR. Phase III trial of irinotecan/cisplatin compared with etoposide/cisplatin in extensive-stage small-cell lung cancer: clinical and pharmacogenomic results from SWOG S0124. J Clin Oncol. 2009;27:2530-5.

51. Hermes A, Bergman B, Bremnes R et al. A randomized phase III trial of irinotecan plus carboplatin versus etoposide plus carboplatin in patients with small cell lung cancer, extensive disease (SCLC-ED): IRIS-Study. . Proc Am Soc Clin Oncol 2008;(abstr 7523).

52. Heigener D, Freitag L, Escbach C et al. Topotecan/cisplatin (TP) compared to cisplatin/etoposide (PE) for patients with extensive disease-small cell lung cancer (ED- SCLC): Final results of a randomised phase III trial. Proc Am Soc Clin Oncol 2008;(abstr 7513).
